# Environmental unpredictability and inbreeding depression select for mixed dispersal syndromes

**DOI:** 10.1186/s12862-016-0638-8

**Published:** 2016-04-05

**Authors:** Jorge Hidalgo, Rafael Rubio de Casas, Miguel Á.Muñoz

**Affiliations:** Instituto Carlos I de Física Teórica y Computacional and Departamento Electromagnetismo y Física de la Materia, Universidad de Granada, Granada, 18071 Spain; Dipartimento di Fisica ’G. Galilei’ and CNISM, INFN, Universitá di Padova, Via Marzolo, 8, Padova, 35131 Italy; Departamento de Ecología, Facultad de Ciencias, Universidad de Granada, Granada, 18071 Spain; Estación Experimental de Zonas Áridas, EEZA-CSIC, Carretera de Sacramento s/n, La Cañada de San Urbano, Almería, 04120 Spain; UMR 5175 Centre Ecologie Fonctionnelle et Evolutive, CEFE-CNRS, 1919 Route de Mende, 34293Montpellier Cedex 05, France

**Keywords:** Bet-hedging, Mixed mating, Mixed dispersal, Selfing, Amphicarpy, Heterocarpy, Environmental noise, Individual based models

## Abstract

**Background:**

Mixed dispersal syndromes have historically been regarded as a bet-hedging mechanism that enhances survivorship in unpredictable environments, ensuring that some propagules stay in the maternal environment while others can potentially colonize new sites. However, this entails paying the costs of both dispersal and non-dispersal. Propagules that disperse are likely to encounter unfavorable conditions, while non-dispersing propagules might form inbred populations of close relatives. Here, we investigate the conditions under which mixed dispersal syndromes emerge and are evolutionarily stable, taking into account the risks of both environmental unpredictability and inbreeding.

**Results:**

Using mathematical and computational modeling, we show that high dispersal propensity is favored whenever environmental unpredictability is low and inbreeding depression high, whereas mixed dispersal syndromes are adaptive under high environmental unpredictability, more particularly if inbreeding depression is small. Although pure dispersal is frequently adaptive, mixed dispersal represents the optimal strategy under many different parameterizations of our models, indicating that this strategy is likely to be favored in a wide variety of contexts. Furthermore, monomorphic populations go inevitably extinct when environmental and genetic costs are high, whilst mixed strategies can maintain viable populations even under very extreme conditions.

**Conclusions:**

Our models support the hypothesis that the interplay between inbreeding depression and environmental unpredictability shapes dispersal syndromes, often resulting in mixed strategies. Moreover, mixed dispersal seems to facilitate persistence whenever conditions are critical or nearly critical for survival.

**Electronic supplementary material:**

The online version of this article (doi:10.1186/s12862-016-0638-8) contains supplementary material, which is available to authorized users.

## Background

Organisms exist in ever-changing environments and need to surmount the challenges posed by external variability. When environmental conditions change unpredictably in time, the appropriate measure of evolutionary success is not the average fitness across generations but its geometric mean [1]. This is because population growth is an inherently multiplicative process that is very sensitive to occasional extreme values [2]. Thus, if organisms cannot accurately predict or detect the most likely environment their offspring will experience, they should hedge their bets by producing a range of progeny phenotypes [3].

Seed dispersal enables plants to distribute their progeny in different environments, minimizing the probability that all of the seeds will end up in a single, unfavorable site. Dispersal is expected to be particularly beneficial when environmental conditions change unpredictably in time [4, 5]. However, dispersal entails a high risk, as dispersers are largely exposed to environmental contingencies. Consequently, it has been posited that mixed dispersal strategies might emerge to accommodate the risks of dispersal while still ensuring some of its benefits. Several authors have supported this view and argued that the production of progeny with contrasting dispersal abilities by a single maternal genotype constitutes an instance of bet-hedging in heterogeneous environments [5–9].

In addition to enabling the sampling of different environments, dispersal away from the maternal site maximizes the probability of individuals encountering mating partners of diverse genetic background. Conversely, locally dispersing individuals are more likely to mate with relatives which would result in the production of inbred progeny [10, 11]. Therefore, the avoidance of inbreeding depression and kin competition is expected to be a primary force in the evolution of dispersal strategies [10–13]. Since kin competition and inbreeding avoidance are not independent, dispersal selection can be summarized as a dynamical balance between the avoidance of inbreeding and the risks of dispersal. However, to the best of our knowledge this has never been explicitly modeled.

**Fig. 1:**
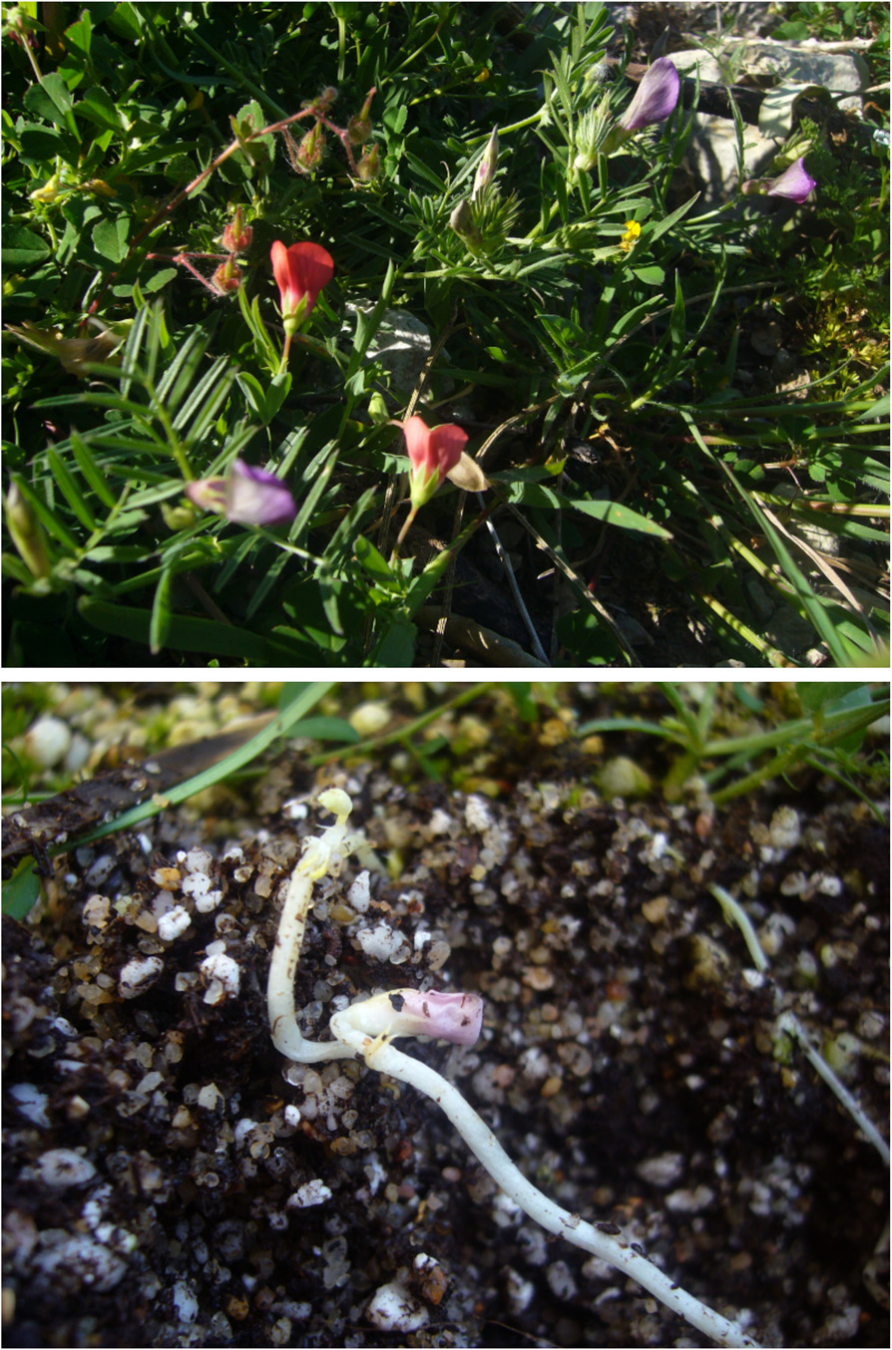
Two examples of amphicarpic plants exhibiting mixed dispersal syndromes. (*Top*) Aerial open-pollinated flowers of *Lathyrus amphicarpos* (*red*) and *Vicia amphicarpa* (*violet*). (*Bottom*) Subterranean self-pollinated flower of *Lathyrus amphicarpos*. Photos by Rafael Rubio de Casas

A paradigmatic example of mixed dispersal syndromes is that of “heterocarpic plants” which produce different types of fruits that vary in their intrinsic dispersal propensity [8, 14–16]. In many cases, the different dispersal phenotypes are produced by flowers that differ in their mating. For instance, there are some plants (called “amphicarpic”) that produce aerial chasmogamous (i.e., open-pollinated) and subterranean cleistogamous (i.e., strictly self-pollinated) flowers. The seeds produced by aerial flowers disperse freely, while the underground seeds are not dispersed [16–19] (see Fig. 1). Although amphicarpy might seem a natural history oddity, this association between open-pollinated flowers and dispersing seeds and selfing flowers and non-dispersing seeds is an extreme example of the positive evolutionary correlation between high dispersal propensity and outcrossing and/or between selfing and limited dispersal, predicted by several authors [10, 11, 20–22].

Assuming that evolution is likely to favor this correlation between dispersal and the mating system, in the present paper we aim to understand the evolution of mixed dispersal strategies using amphicarpic plants as reference system. Because both the risk of environmental change and inbreeding are expected to shape dispersal strategies, we deem a theoretical framework that accommodates the two processes to be a useful contribution to the study of dispersal evolution. Our approach differs from previous studies of the correlation between dispersal and the mating system –most notably from that of [17]– in that it explicitly incorporates both environmental and genetic costs.

More specifically, here we develop computational individual-based models in which organisms live in a (discretized) two-dimensional non-saturated space. Our models are inspired by annual plants, i.e. by organisms with non-overlapping generations, so all individuals die and are removed from the community at the end of each reproductive cycle (year). During their lives, individuals produce seeds that can either disperse or not with a probability controlled by parameter *α* that represents the. The resulting seeds are established or not depending stochastically on changing environmental conditions as well as on their level of inbreeding. As a first approach, we consider a simple, reductionist case that can be assimilated to amphicarpy: plants produce two types of seeds that differ both in their propensity for dispersal (dispersing versus non-dispersing) and in their level of inbreeding (outcrossing versus selfing). In this case, we posit a perfect association between dispersal and the mating system, i.e. dispersers outcross while non-dispersers are the only ones suffering from inbreeding effects (we have also analyzed a variant of the model in which this perfect association is relaxed). In our approach, a maximum of one plant survives per site from one generation to the next, while some sites may become empty. After many generations, the system reaches a stationary density of plants, *ρ*, which can be zero (i.e., result in extinction) if the population is not viable. In such cases, we measure the average extinction time, *T*. By measuring *ρ* and *T*, we can determine which level of dispersal propensity *α* leads to the best possible outcome, i.e. maximal density and/or longest extinction times. As a second step, we implement evolutionary algorithms in which each plant is equipped with its own value of *α*, which is transmitted with small mutation to its offspring. This type of genetic algorithm selects automatically the best parameter (dispersal propensity in our case) values as evolutionary stable because favored individuals propagate rapidly and do not allow other solutions to spread in the population.

The results of these models show that the specific strategy that is selected for depends on the interplay between inbreeding depression and environmental variability, although mixed dispersal seems to be more favorable and robust under many circumstances.

## Methods

### Model implementation

**Fig. 2:**
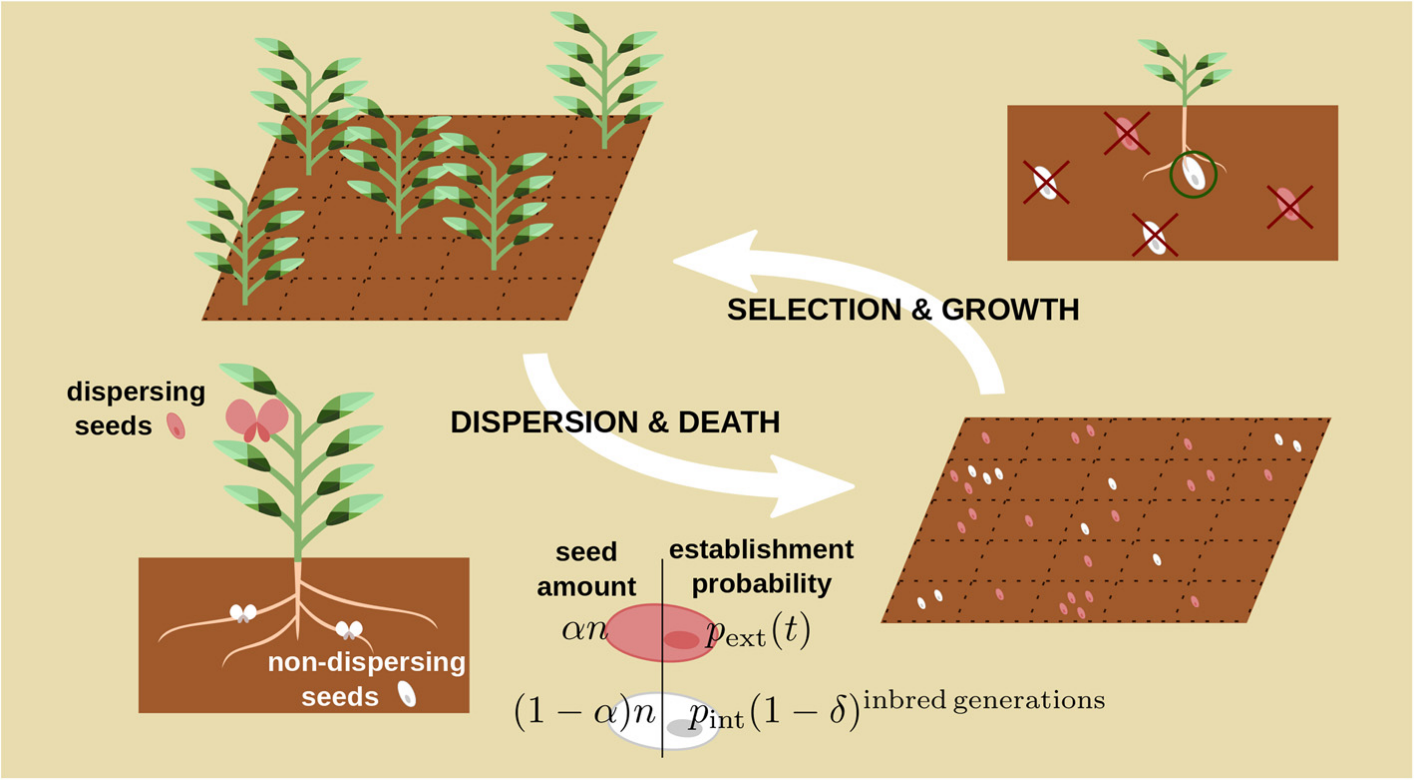
Community of plants with mixed dispersal phenotypes. Each plant is located at a cell of a square lattice of size *L*×*L*. Each individual plant produces the same fixed number of seeds, *n*; seeds can be of two different types: “dispersing” and “non-dispersing”, marked in red and white respectively in the sketch. Each produced seed is external/dispersing with probability *α*, or internal/non-dispersing with probability, 1−*α*, where *α* is the dispersal propensity parameter. After reproduction, all adult plants are removed from the community. Regardless of their origin, dispersing seeds can randomly arrive to any cell in the lattice where they get established with probability *p*_ext_(*t*) which is environment-dependent. On the other hand, non-dispersing seeds can only establish themselves at the maternal location or in its adjacent lattice cells. Then, for cells with more than one established seed, one of these is chosen at random and the rest die. The establishment probability *p*_int_ of non-dispersing seeds is assumed to be independent from environmental variability (and thus, it does not depends on time). In the simplest case, dispersing seeds are produced by outcrossing, whereas non-dispersing seeds are the product of selfing. Thus, their quality, *q*, is reduced after each inbreeding event by a penalization factor *q*→(1−*δ*)*q*. In our formulation, inbreeding depression is approximated in a manner that can be assimilated to the interaction among many slightly deleterious alleles that affect the trait independently (e.g., [36]). The number of alleles determining inbreeding is assumed to be *n*→*∞*. Therefore, it is always proportional to homozigosity and accumulates multiplicatively with inbreeding events. Outcrossing events are expected to eliminate homozigosity and to reset *δ*=0 and *q*=1, so dispersing seeds are always assumed to not have any inbreeding depression. We also study a more general scenario in which selfing is not restricted to a specific dispersal syndrome, but in which inbreeding affects the quality of seeds produced by individuals that mate with relatives (see Additional file 1: S1). In this case, inbreeding is proportional to the kinship between mating partners. This generalized model is less restrictive in that there is no assumption of a perfect association between dispersal and mating, but leads to qualitatively similar conclusions

We present a simple and parsimonious individual-based model in which a population of plants develops in time through the processes of birth, reproduction, competition, and death (Fig. 2). Each individual/plant lives at a fixed site on a two-dimensional square lattice of size *L*×*L*. Periodic boundary conditions are assumed to minimize contour effects. In order to account for local competition for resources and space, occupancy is restricted to a maximum of one plant per site, and the lattice is typically not fully occupied.

At each discrete time-step *t* –which represents a reproductive cycle, i.e. one year in the case of annual plants– all occupied lattice sites are emptied, i.e. generations are assumed to be non-overlapping, and new plants emerge from existing seeds following some dynamical rules that we specify in what follows. Each individual plant produces the same, fixed, number of seeds, *n*, but these can be of two different types/morphs: “dispersing” and “non-dispersing”, respectively. Dispersing seeds travel to arbitrarily distant sites –for simplicity, we assume that they can end up randomly in any location within the lattice– whereas non-dispersing seeds can only stay in the maternal or adjacent sites. Importantly, we do not address spatial heterogeneity explicitly, and all sites away from the maternal one are assumed to be equivalent (i.e., sites can be of two classes local or “internal”, occupied by non dispersing seeds or “external”, where dispersing seeds might land). The relative fraction of the two morphs is modulated by the dispersal propensity parameter *α*: with probability *α*, each of the produced seeds is dispersing, or, complementary with probability 1−*α*, non-dispersing. Initially, we take *α* as a constant, while allowing for variability in other model parameters (e.g., the degree of inbreeding depression and environmental variability). In a second step, we study the case in which *α* is dynamically self-tuned in the community.

Even if, for the sake of simplicity, we assumed that the reproductive output –i.e. the total number of seeds produced by each individual plant– is constant, fitness differs among maternal plants because the probability of establishment is constrained by environmental conditions and inbreeding depression and thus the actual contribution to the next generation is individual-dependent.

Each morph follows a different type of dynamics:

- Dispersing seeds establish at a randomly selected site with a probability *p*_ext_(*t*)∈[0,1] –which is a fluctuating random variable assumed to depend upon changing environmental conditions, such as rain, temperature, predation, etc. and to be equal at each time step for all sites in the lattice– or, alternatively, they can be lost with complementary probability 1−*p*_ext_(*t*). For simplicity, we take *p*_ext_(*t*) to be an uncorrelated random variable –freshly extracted at each discrete time step– with uniform distribution in $[\bar {p}_{\text {ext}}-\sigma, \bar p_{\text {ext}}+\sigma ]$ with the constraint that $\sigma < \min (\bar p_{\text {ext}}, 1-\bar p_{\text {ext}})$.
- Non-dispersing seeds are assumed not to be influenced by temporal environmental variability, but to suffer from inbreeding depression. In particular, individual plants are equipped with an individual *quality* parameter *q*, which is inherited by the seeds they produce. The actual establishment probability of non-dispersing seeds is *q*×*p*_int_, with maximum *p*_int_. The quality *q* modulating the establishment probability is set initially to *q*(*t*=0)=1 for all individuals in the community, but it is reduced by a factor (1−*δ*)<1 every time there is selfing (i.e., a reproductive event resulting in a non-dispersing seed), or instead, it is reset to *q*→1 by outcrossing, i.e., when a plant ensues from a dispersing event.

After all reproductive and possible establishment events, at each timestep (year) only one seed is randomly selected (with homogeneous probability) for reproduction at each cell with one or more established seeds, while the rest are removed from the community; if there is no seed in a cell, it is left empty.

### Model extensions and robustness

The described model is a parsimonious one, restricted to the simplest syndrome (perfect and immediate association between dispersal and mating) and limited by important assumptions: temporal environmental variability affects only dispersing seeds and the genetic load is completely eliminated after a single outcrossing event. To test the robustness of our results against these restrictions, we also explored variants of the model in which each of them has been relaxed. These results are presented in detail in the Additional file 1. In the work shown as Additional file 1: S1 the model was modified to eliminate the perfect association between dispersal and mating. In this variant, plants still produce two types of propagules, but inbreeding depression is derived as a function of the proximity to relatives. In this case, we assume that mating occurs between individuals that are spatially close and inbreeding depression ensues from the mating between individuals that are genetically related. In Additional file 1: S2 we analyzed another variant of the model in which recovery from inbreeding depression is only partial and not perfect after a single outcrossing event. In this case, the “genetic quality” of seeds has its own dynamics in time depending on the type of mating (see Additional file 1: S2). Lastly, in Additional file 1: S3, we present a more complex approach in which the influence of environmental noise on the probability of establishment of non-dispersing seeds is also taken into account. Albeit all these extensions modify the results in different ways, the main conclusions are always similar and match those derived from the more basic, initial model. Consequently, and for the sake of simplicity, this is the focus of the analyses presented in the main text.

### Computer simulations and analyses

Given that all our computer simulations are run considering finite and closed populations, extinction is always possible –even in cases with a relatively high stationary density (i.e., big population sizes)– as a consequence of demographic fluctuations, and once all individuals have disappeared from the community, the system reaches a stationary state and remains quiescent indefinitely. States with a non-vanishing stationary density are thus not truly stationary, fluctuations may always lead them to extinction; for this reason the term “quasi-stationary” is used in reference to steady states of realizations that have not fallen into the quiescent (also called “absorbing”) state [2].

Extinctions can then be classified in two different categories:

- *Deterministic* extinctions, which occur unavoidably after a given characteristic time (which grows slower than linearly with system size). When this occurs, the system is said to be in the “absorbing” phase.
- *Accidental* extinctions, which correspond to catastrophic demographic fluctuations, the probability of which rapidly (exponentially) decreases with system size. These only occur –as a result of finite population size– when the system is in its “active” phase, and is also characterized by a non-vanishing (quasi)stationary density of individuals *ρ*≠0.

In order to determine –for a given set of parameter values– in which phase lies the system, we measured computationally the mean-extinction time *T* as a function of the linear system size *L*. *T* grows exponentially or algebraically with *L* in the active phase, while it converges asymptotically to a constant value in the absorbing one. Additionally, to measure the quasi-stationary density in an efficient way, we re-activated any iteration reaching the quiescent state (i.e., extinction) by setting it to a very small but non zero density (i.e., re-introducing by hand a few individual plants in the community). In particular, we verified that reseeding the system with introducing any number of plants between 2 and 10 did not significantly affect the results. The reintroduced individuals had in every case the same dispersal propensity *α* of the last survivor before extinction. Albeit admittedly ad-hoc, this computational trick leads to very similar results than other more sophisticated exact methods [23] (in fact, our method can lead to slight numerical differences with respect to more accurate methods, but we have explicitly verified that these remain non-significant for the phenomenology presented here).

Our working hypothesis is that, in temporarily unpredictable environments or when inbreeding depression is significant, mixed dispersal strategies (0<*α*<1) might lead to higher individual fitness than either of the single phenotype syndromes. To test this, we first developed a preliminary study of the stationary density *ρ* for different values of the dispersal parameter *α*, while keeping all other parameters fixed. We find that for each choice of parameters, there exists a specific value *α*^∗^ for which the population density is maximized. If this optimal value takes an intermediate value between 0 and 1, the optimal strategy is mixed.

Additionally, we implemented an evolutionary approach in which *α* is not a constant, kept fixed across the whole population and across generations but an inheritable trait/variable. In particular, we defined a genetic algorithm similar to that of [24, 25] in which each individual has its own dispersal syndrome, as encoded in a specific value of its parameter *α*; this value is transmitted to its progeny with a stochastic (Gaussian distributed) variation of zero mean and *ν* standard deviation. In biological terms, *ν* can be understood as the rate of mutation, recombination and other sources of variation in heritable traits across generations (referred to as mutation rate hereafter). In this evolutionary version of the model, individuals with low fitness tend to become extinct, while the space they leave empty becomes progressively occupied by fitter individuals (individuals with a higher probability of establishment). The outcome of this evolutionary dynamic is a population with some averaged (quasi)steady-state density, *ρ*, and a well-defined average value of *α* across the community, that we call $\bar {\alpha }$, and some variance around these mean values. Admittedly, this modeling exercise does not represent a realistic evolutionary process. However, it provides an effective and dynamical approach to study how individuals in the population self-optimize their dispersal strategies across generations as a result of competition and mutation.

## Results

### Single phenotype cases: *α*=0,1

As a first step, we studied the behavior of the population when it exhibited any of the two single-phenotype syndromes, i.e. when all the individuals presented the same, homogeneous dispersal morph, in our formulation expressed by a fixed dispersal propensity parameter of either *α*=1 or *α*=0, for purely dispersing and non-dispersing populations, respectively. Then, we computed through numerical simulations (starting from a fully occupied lattice) the stationary population density, *ρ*, as a function of the parameters determining the probability of establishment, environmental unpredictability and inbreeding depression: $\bar p_{\text {ext}}$, the probability of establishment away from the maternal site and *σ* (environmental heterogeneity) for the purely dispersing morph (*α*=1) and *p*_int_, the probability of establishment within the maternal site and *δ* (inbreeding depression) for the non-dispersing morph (*α*=0). In most of what followed, we fixed for convenience the original establishment probabilities for both types of seeds to be identical, $p_{\text {int}}=\bar {p}_{\text {ext}}$, but our main results do not change, at least qualitatively, for asymmetric values of establishment probabilities (note that identical arithmetic means do not imply identical geometric means, which are more relevant in the long term; see Additional file 2: Appendix).

**Fig. 3:**
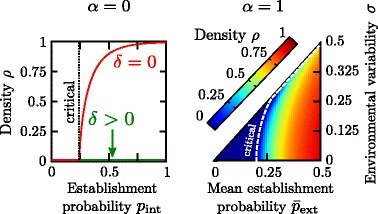
Stationary density of single phenotype syndromes. Both single-phenotype dispersal syndromes, *α*=0 (*left*) and *α*=1 (*right*), exhibit a phase transition between an *absorbing* phase where the population always becomes extinct, and an *active* phase, with a sustained non-vanishing stationary density *ρ*. For the non-dispersing syndrome (*left*) the population always becomes extinct with non-null inbreeding depression (*δ*>0, green curve), while there is a phase transition at *p*_int_≃0.24 if *δ*=0 (red curve). In the dispersing syndrome (*right*), the critical point increases as a function of the degree of environmental variability, *σ*>0, i.e. variability is detrimental to population density, expanding the absorbing phase (dark blue region) at the expense of the active one. The critical line has been computed with the analytical approach described in the Additional file 2: Appendix for an infinite system, *L*=*∞*. To compute the stationary densities, we iterated for 10^4^ generations, and averaged over the last 10^4^/2 steps; averages over at least 100 independent realizations were performed. Parameters are set to *L*=100 and *n*=5

The left panel of Fig. 3 shows the stationary density *ρ* for the pure non-dispersing syndrome (*α*=0). In the absence of inbreeding depression (*δ*=0), there is a critical point located at $p_{\text {int}}^{c} \simeq 0.24$. However, for any non-zero inbreeding depression (*δ*>0), the purely non-dispersing phenotype *α*=0 is doomed to extinction regardless of the establishment probability *p*_int_.

Figure 3 (right panel) illustrates the stationary population density for the dispersing syndrome (*α*=1) for different choices of the environmental parameters $\bar p_{\text {ext}}$ and *σ*: a continuous phase transition separates an absorbing phase (region above the dashed line) in which the population becomes (deterministically) extinct and an active one, in which a non-trivial steady state is reached (region below the dashed curve). In the absence of temporal environmental variability (i.e. *σ*=0) the critical point at which the phase transition occurs lies at a *critical* establishment probability $\bar {p}_{\text {ext}}^{c}(\sigma =0)=1/n=0.20$ (i.e. when persistence is ensured by the production on average of one viable offspring by each maternal individual): large probabilities *p*_ext_ entail non-trivial steady states, and small ones lead ineluctably to extinction. Similarly, as *σ* increases larger establishment probabilities are needed to sustain a viable population.

Measurements of averaged extinction times were used to confirm the location of the critical lines in both cases. Furthermore, the critical lines can be calculated analytically (Additional file 2: Appendix).

Although the two single-phenotype cases are overly simplistic and results are somewhat predictable, they provide a useful reference to frame the dynamics of mixed strategies.

### Mixed dispersal syndromes

**Fig. 4:**
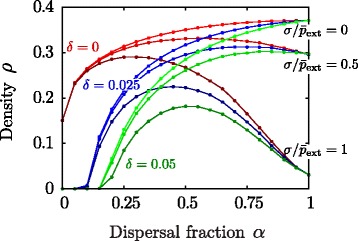
Stationary density for the mixed dispersal syndromes as a function of the dispersal propensity parameter ***α***. Parameters of the single phenotype syndromes are set to $p_{\text {int}}=\bar p_{\text {ext}}=0.25$ and *n*=5. Linear system size *L*=100 (total size *N*=10000). Stationary density for three different values of inbreeding depression (represented by red, green and blue curves, respectively) and environmental variability (darker shades indicate higher environmental unpredictability; labeled at *α*=1). Both *δ* and *σ* tend to reduce the population density in the pure strategies (*α*=0 or *α*=1, respectively) but, remarkably, relatively large densities can be attained by populations with mixed syndromes even in the presence of inbreeding depression and environmental unpredictability. Although the specific values of *δ* and *σ* are not intended to be biologically realistic, change in these parameters illustrates qualitatively the consequences of different genetic and environmental costs. Note that points at parameter values *α*=1 and *σ*=0.25 correspond to the absorbing region (see the calculation $\bar p_{\text {ext}}^{c}$ in the Additional file 2: Appendix for $\sigma =p_{\text {ext}}^{c}$) however measurements of the (quasi)stationary density give small positive values, which decrease to zero for larger system sizes (not shown)

As a second step, we explored how the stationary density, *ρ*, changes as a function of the dispersal propensity parameter *α* for various environmental and inbreeding conditions (i.e. for different values of *σ* and *δ*). Results are summarized in Fig. 4 (again, simulations were run fixing $\bar p_{\text {ext}}=p_{\text {int}}$). First, it can be noticed that, in the absence of environmental variability (for *σ*=0), the stationary density grows monotonically with *α*, indicating that the purely dispersing syndrome leads to higher population densities. This might seem surprising, especially in the case *δ*=0 as both strategies are controlled by the same establishment probability. However, given that, in general, the system is not saturated, dispersal tends to be favored because it enables the colonization of new sites, ultimately leading to larger population densities.

As the value of *σ* increases, the density attained by populations exhibiting the purely dispersing syndrome decreases whereas populations with mixed syndromes (intermediate values of *α*) reach larger stationary densities than the pure dispersal strategy. The relative advantage of mixed syndromes is even more conspicuous when inbreeding depression is also significant (*δ*>0). For instance, for *δ*=0.025 and *σ*=0.125, the curve is non-monotonous and has a parabolic-like shape, with local minimal densities at *α*=0 and *α*=1 and with a maximum at *α*^∗^≈0.7.

Moreover, we observe a broad region in parameter space ($\delta >0,\sigma \gtrsim 0.2$) where the population becomes extinct when individuals spread through either of the single phenotype syndromes but it can survive with non-vanishing densities when the dispersal syndrome is mixed.

#### Extinction times

**Fig. 5:**
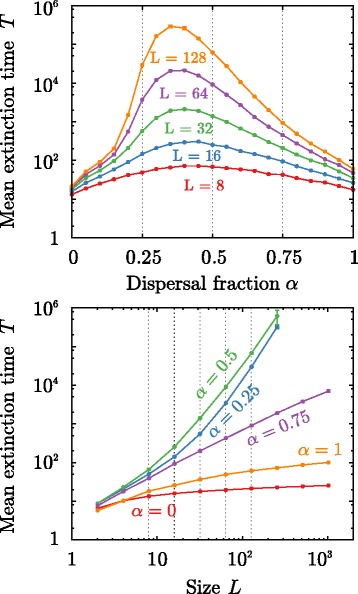
Mean extinction time, *T*, for mixed dispersal syndromes as a function of the dispersal fraction *α* (*top*) and of linear system size *L* (*bottom*). Parameters of the single phenotype syndromes are set to $p_{\text {int}}=\bar p_{\text {ext}}=0.25$, *n*=5, *δ*=0.05 and *σ*=0.25, corresponding to the absorbing phase (see Fig. 3). Each point was computed averaging over 10^3^ realizations of the simulations. Most error bars are smaller than point size. Dashed lines have been included to facilitate comparisons across panels. (*Upper panel*) Extinction time is always maximum when 0.25<*α*<0.5 and converges to *α* ^∗^≈0.4 for large sizes of the system). (*Lower panel*) Populations exhibiting any of the single-phenotype strategies (*α*=0 and *α*=1) are in the absorbing phase, as indicated by the downward curves on the log-log scale, i.e., their extinction is unavoidable (deterministic extinction). Conversely, intermediate values of *α* lead to extinction times that grow exponentially with system size, and thus maintain populations in the active phase

To confirm the positive effect of mixed dispersal strategies, we performed the following computational experiment: we adjusted the values of environmental heterogeneity and inbreeding depression (the free parameters of our model) to be *σ*=0.25 and *δ*=0.05 to make both purely dispersing and purely non-dispersing syndromes nonviable on the long term, leading ineluctably to extinction. Starting from a fully occupied system, *ρ*=1, we computed the mean extinction time for different values of *α* and various linear system sizes *L*. Results are shown in Fig. 5. Figure 5 (upper panel) shows the averaged extinction time *T* as a function of *α* for system sizes *L*=8,16,32,64 and 128. In all cases, larger systems have longer extinction times. However, although *T* barely varies for single phenotype syndromes (*α*=0,1), extinction times rapidly increase for populations exhibiting mixed dispersal syndromes (e.g. *α*=0.5). Figure 5 (lower panel) illustrates the extinction time as a function of the system size for values of the dispersal parameter *α*=0,0.25,0.5,0.75 and 1. While single dispersal syndromes (*α*=0,1) exhibit a slower-than-linear dependency (concave function on a log-log scale), extinction times increase exponentially for mixed dispersal syndromes (convex function on a log-log scale). In biological terms this means that, even in cases where a population consisting only of purely dispersing or purely non-dispersing individuals would go extinct in a relatively short time because of environmental or genetic constraints, mixed dispersal can facilitate population persistence, enabling survival for exponentially large times.

Finally, we used a mathematical calculation to understand how mixed dispersal syndromes facilitate higher population densities and much longer extinction times. These analyses are described in detail in the Additional file 2: Appendix. In a nutshell, we computed –in the simplest possible scenario– the averaged exponential growth rate for small population densities, *G*, as a function of dispersal propensity, *α*. In this case, the (non-linear) effects of competition and saturation can be safely neglected, rendering the calculation amenable to exact analytical solutions. The sign of *G* determines whether the population tends to shrink and disappear (*G*<0), or, instead, to grow and survive (*G*>0). Our results show that *G*(*α*) is a non-linear function such that, even for parameter values for which both single dispersal syndromes would lead to extinction (*G*(*α*=0,1)<0), some mixed syndromes can result into a positive growth rate (*G*(*α*)>0), allowing for long-term persistence [26]. Thus, even if for a specific, limited case, our mathematical analysis fully supports the computational findings above.

#### Evolutionary stable strategy (ESS)

In the evolutionary version of the model, the dispersal propensity parameter *α* evolves as a consequence of the dynamics of the system. Here, each individual plant has its own value of *α*, which is transmitted to its progeny (i.e. to each seed, then to the new plants) with a small Gaussian mutation with zero mean and standard deviation *ν*, imposing that *α*=0 (resp. *α*=1) whenever *α*<0 (*α*>1).

Initially (at time *t*=0), all plants are considered to have *α*=1/2. At each generation, *P*(*α*,*t*) is dynamically modified and, eventually, achieves a stationary shape *P*(*α*,*t*→*∞*), identified with the dispersal evolutionary stable strategy (ESS). At this point, we compute the stationary density of the population and the mean-value of *α* from such a distribution, that we call $\bar {\alpha }$. Runs that led to accidental extinction were re-activated with a few individuals (any number from 2 to 10) with a value of *α* chosen at random from those of the generation immediately preceding extinction. After verifying that stationary values of density *ρ* and *α* were largely independent of *ν* (if *ν* is sufficiently small) we fixed *ν*=10^−3^.

**Fig. 6:**
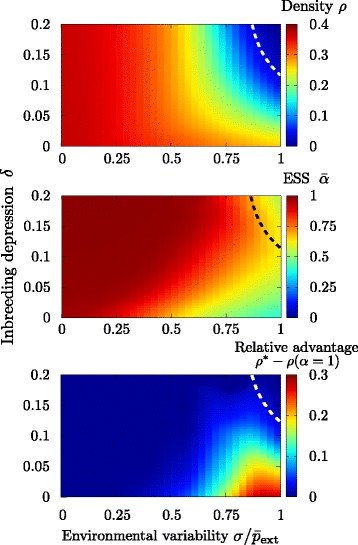
Optimal strategy. The mean dispersal propensity parameter *α* is self-tuned dynamically in a community of individuals through evolutionary dynamics, based on the genetic algorithm of [24, 25]. (*Upper panel*) Color plot of the stationary density as a function of the inbreeding depression *δ* and the environmental variability *σ*; the dashed line represents the critical line separating absorbing and active phases in the quasi-stationary ensemble (i.e., the system is not allowed to go fully extinct) and is established as the parameter combinations that ensure a stationary density =1 *%*. Although individual plants have the possibility of developing mixed strategies, the population always becomes extinct whenever both *δ* and *σ* are high; i.e. under very adverse conditions. (Middle panel) Average value of *α* across the population in its steady state. The dispersing syndrome is favored whenever the environmental variability is low *σ*<0.05 and/or inbreeding depression is high (red region). The ESS corresponds to mixed dispersal when environmental variability is significant (orange to green region). The parameter space in which the ESS corresponds to predominantly non-dispersing syndromes is very narrow (hardly visible dark blue region in the lower right hand corner) and requires inbreeding depression to be negligible and environmental variability to be large. (*Lower panel*) Relative advantage of the mixed dispersal syndromes: stationary density of the optimal mixed dispersal syndrome, *ρ* ^∗^ (obtained from the genetic algorithm), minus the density for the fixed dispersal syndrome. Parameters have been set to *L*=100, $p_{\text {int}}=\bar p_{\text {ext}}=0.25$, *n*=5 and *ν*=10^−3^, and averages are performed over the last 10^5^/2 steps in 10 independent simulations iterated for 10^5^ generations

Results for the mean density and mean value of *α* are shown in Fig. 6 (upper and middle panel, respectively). Populations can only be viable (i.e., stay within the active phase to the left of the dashed line representing the critical transition) under certain combinations of environmental variability and inbreeding depression. The ESS always leads to non-saturated populations (*ρ*<0.4) and is provided by dispersal syndromes that vary from mixed to fully dispersing ($\bar {\alpha }$ in the interval [≈0.35,1] in the active phase). These solutions have relatively small standard deviations (below 0.04) around their mean values, indicating that there is little heterogeneity in dispersal strategies across the population in the steady state. Remarkably, the values of $\bar {\alpha }$ are very close to the optimal values of *α*^∗^ that result in maximum population densities. In summary, our evolutionary models corroborated the optimality approach and indicated that selection should always favor populations that are either strictly dispersing or have mixed dispersal.

It is worth noting that, in principle, optimal values of $\bar {\alpha }$ can be calculated even for populations in the absorbing phase. However, given that a true steady state does not exist in this case, the computed results could be influenced by the initial conditions imposed and the re-seeding method. Although a detailed investigation of the dynamics of extinction could be interesting, the analysis of the phenomena within the absorbing region is beyond the scope of the present work.

As a final step, we tried to quantify the relative evolutionary advantage provided by mixed dispersal syndromes with respect to pure phenotypes. This was estimated as the difference between the steady state density of a population exhibiting the optimal dispersal strategy (ESS) and the maximum density attainable by a population exhibiting a purely dispersing syndrome: *ρ*^∗^−*ρ*(*α*=1). No comparison with the single non-dispersal phenotype was computed because this strategy always leads to extinction, *ρ*(*α*=0)=0, for any non-zero value of inbreeding depression. Results for different environmental and inbreeding parameters values, *σ* and *δ*, are reported in the lower panel of Fig. 6. The increase in population density provided by the emerging optimal mixed syndrome is much higher in the lower right part of the Figure, i.e. for relatively small values of inbreeding depression (*δ*<0.05) and relatively large values of the environmental variability *σ*>0.2.

## Discussion

The results derived from our models showed that dispersal syndromes can be the direct outcome of the interplay between inbreeding depression and temporal environmental variability. Depending on the specific strength of these two forces, the optimal dispersal syndrome can either ensure high dispersal propensity, very limited dispersal, or a mixed situation in which individuals employ a combination of both strategies.

According to our analyses, pure populations of non-dispersers can only be viable on large timescales (i.e., reach non-trivial steady-state densities *ρ*>0) in the complete absence of inbreeding depression (*δ*=0). Similarly, temporal environmental variability reduce the steady state density of pure dispersing populations but, contrary to non-dispersers, viable populations of dispersers can exist under a wide range of environmental uncertainty. Under rather generic conditions, populations with a pure dispersing syndrome tended to perform better than pure non-dispersing populations. This result was not unexpected, as in the absence of environmental variability our model penalized non-dispersal through inbreeding depression. However, it highlights the role of inbreeding depression in the evolution of dispersal, in agreement with the results obtained by other authors [10, 11, 22].

One of the goals of this work was to investigate whether it is possible for mixed dispersal strategies to emerge even in relatively spatially homogeneous environments. Our models did not include fine-grained spatial heterogeneity, which is expected to favor the emergence of limited or mixed dispersal syndromes [27]. Moreover, we did incorporate a high level of temporal heterogeneity, which should select for dispersal [28]. In spite of these limitations, our results showed robustly that mixed dispersal can be favored under a wide variety of conditions.

Generally speaking, optimal dispersal strategies appear to be represented by mixed syndrome in which plants produce simultaneously dispersing and non-dispersing seeds; populations with dispersal fraction 0.25<*α*<0.75 attained higher densities and were viable for longer whenever environmental conditions were highly fluctuating (i.e., for any *σ*≥0.2). Moreover, mixed dispersal appears to enable positive growth rate and long term survival even under conditions for which both single dispersal phenotypes would lead to extinction. This is congruent with the findings of Jansen and Yoshimura [29], who showed that populations can persist in an environment consisting of two sink habitats if offspring are randomly distributed over both of them. Our results support this idea and indicate that mixed syndromes can be advantageous due to the benefits of bet-hedging through multiple co-existing complementary strategies.

In all of our computational analyses, patch size was finite, and thus populations could go extinct –following stochastic demographic collapse– relatively easily. More importantly, according to our models, population extinction was unavoidable, regardless of patch size, whenever inbreeding depression or extreme environmental variability occurred. In particular, dispersing syndromes led to accelerated extinction if the population was subject to adverse environmental conditions for several generations. These situations might or might not take place in real-world scenarios, where meta-population dynamics can buffer the effect of local extirpations through migration from other sources [30, 31]. However, our results showed that even in the absence of immigration, populations exhibiting mixed strategies had significantly longer extinction times, and these grew very fast with patch size. This result is likely contingent on temporal autocorrelation of the environment, and if the latter is positive, extinction is expected to be faster under unfavorable conditions for any given phenotype. However, even if no phenotype can guarantee unlimited survival in finite patches, expected extinction times are drastically enhanced if organisms display mixed syndromes.

The dynamical/adaptive version of our model also supported the hypothesis that populations with mixed syndromes are more resilient. In this version of the model, dispersal propensity was not fixed but rather dynamically self-organized to its optimal value. Results showed that mixed syndromes appeared to provide the highest population densities and the longest population life-spans. In our formulation, non-dispersing had an intrinsic penalization for any non-null value of inbreeding depression. In spite of this relative advantage of the dispersing phenotype, pure dispersal was found to be the evolutionary stable strategy only if environmental unpredictability was low, especially if inbreeding depression was also low. Other authors have predicted that mixed dispersal is adaptive in heterogeneous environments [8, 27, 32]. Our models indicated that this is indeed the case, but that the optimal dispersal strategy is also contingent on inbreeding depression, and that if the latter is significant, pure dispersers might have an advantage even in heterogeneous environments.

Even though both temporal environmental and genetic costs influence the evolution of dispersal, environmental variation appears be specially relevant for the emergence of mixed dispersal strategies, particularly when the requirement of a perfect association between mating and dispersal was released. According to our initial model, mixed syndromes provided the ESS under high temporal environmental unpredictability and/or low inbreeding depression. Moreover, under those same conditions they had a significantly higher fitness than any other alternative phenotype. When we generalized the model to release the association between the mating system and dispersal, results showed an even wider region in which mixed syndromes were favored, mostly due to the lesser influence of inbreeding depression. This result might be seen as contradicting the findings of the model put forward by [17]. These authors showed that mixed mating/mixed dispersal can become the ESS when each type of propagule provides a clearly different advantage; higher establishment in the case of non-dispersing, inbred seeds and lower sibling competition in the case of outbred, dispersing seeds, and these predictions are independent of environmental variability. In our models, sibling competition and other kin selection mechanisms are implicitly incorporated into the inbreeding depression term (i.e., they can be regarded as deleterious consequences of being in close proximity to kin), while environmental variability affects the probability of establishment of dispersing seeds alone. Thus, our model partially corroborates Schoen and Lloyd’s findings [17]; mixed syndromes can be beneficial when there are simultaneous costs to dispersal and coexistence with kin. However, our predictions deviate from Schoen and Lloyd’s in that we anticipate a relatively wide range of conditions under which mixed dispersal can be selected for, and predict that, in the (near) absence of deleterious interactions with kin, costs of dispersal might be enough to select for mixed dispersal.

The purely non-dispersing syndrome was never found to provide an ESS. Our initial formulation imposed a perfect association between dispersal and mating, which might have penalized non-dispersers by making them necessarily more inbred. However, releasing this assumption did not increase the viability of non-dispersal. Although relatively low values of *α* were observed under high environmental variation, they were always ≥0.25. This is somewhat surprising, as plants with monomorphic non-dispersal syndromes do exist. For instance, there are several taxa that produce all their seeds underground (i.e., geocarpic) such as the peanuts (*Arachis* spp.), *Trifolium subterraneum* or *Macrotyloma geocarpum* and non-dispersal has been shown to be an ESS by different models [27, 33]. According to these models, non-dispersing phenotypes are adaptive whenever the probability of establishment away from the maternal site is lower than within the maternal site (i.e., whenever there is local adaptation). We have not explored in detail these scenarios in our models but we can anticipate that non-dispersal syndromes could emerge as ESS by considering the extreme case in which $p_{\text {int}} \gg \bar p_{\text {ext}}$ and inbreeding depression is negligible, *δ*≈0 or if the models allowed for purging or included a transmission advantage of selfing [34]. Clearly, the adaptive value of limited dispersal needs further investigation, even though these sort of phenotype is exceptional in nature [35].

Our conclusions might have been of course biased by the assumptions of the models, some of which are particularly constraining. Besides the perfect association between mating and dispersal which implies that only non-dispersing seeds are affected by inbreeding, our initial model also assumed (i) complete release from genetic depression after a single outcrossing event, and (iii) an absolute buffering of non-dispersing seeds from environmental change. To ensure the robustness of our results, we developed extensions of the model releasing each of these assumptions. These variants are included as Additional file 1 and show that the constraints do not seem to affect the main results significantly. In all cases, the optimal dispersal syndrome was determined by a trade-off between the pressures imposed by environmental heterogeneity and those imposed by inbreeding, with mixed dispersal syndromes representing the ESS in a broad region of the parameter space.

Finally, it is important to note that the way in which we implemented evolutionary dynamics into our calculations provide just a simple way to define and measure ESS, and may be unrealistic. For instance, we assumed that both types of reproduction and dispersal have the same costs for the maternal plant and that the two types of propagules produce identical individuals in the absence of inbreeding depression or environmental variability. However, the empirical and theoretical literature have shown that non-dispersing and dispersing propagules require different resource allocation from the mother plant and can produce different progeny [16]. Moreover, it is possible that mixed syndromes are not easy to develop. It could be expected that the mutations necessary to generate a mixed system, i.e., mutations from *α*=0,1 to *α*≠0,1 would have high pleiotropic costs and be hard to gain. This sort of functional polymorphism entails the coexistence of two distinct phenotypes within a single individual, each phenotype comprising a suite of traits (e.g., flower and fruit tissues, architectural traits, etc.) with their own development and maintenance particularities. However, a biologically plausible model able to incorporate these additional complexities is beyond the scope of the present work.

## Conclusions

Our results show that, although dispersal can be selected for under a wide range of conditions, mixed dispersal syndromes should be favored by low inbreeding depression if non-dispersing entails less variability in the probability of establishment. Moreover, mixed syndromes seem to ensure the viability of populations for longer periods of time, particularly when environmental and inbreeding risks are high.

## Ethics

Not applicable.

## Consent to publish

Not applicable.

## Availability of data and materials

Not applicable.

## Additional files

Additional file 1 Supplementary Information. Generalizations of the model: (**S1**) absence of an explicit association between dispersal and mating, (**S2**) partial recovery with outcrossing and (**S3**) sensitivity of the non-dispersing syndrome to environmental fluctuations. (PDF 1142 kb)

Additional file 2 Appendix. Analytical approach for the model. (PDF 216 kb)
