# Supplementary material for: Environmental unpredictability and inbreeding depression select for mixed dispersal syndromes
Source: BMC Evol Biol. 2016 Apr 5;16:71. doi: 10.1186/s12862-016-0638-8 (PMC4820946; doi:10.1186/s12862-016-0638-8)
Supplement: Additional file 1 — Supplementary Information. Generalizations of the model: (S1) absence of an explicit association between dispersal and mating, (S2) partial recovery with outcrossing and (S3) sensitivity of the non-dispersing syndrome to environmental fluctuations. (PDF 1142 kb) [file 12862_2016_638_MOESM1_ESM.pdf]

RESEARCH

Supporting Information for: Environmental unpredictability and inbreeding depression select for mixed dispersal syndromes

Jorge Hidalgo[1,2], Rafael Rubio de Casas[3,4,5*] and Miguel Á. Muñoz[1]

*Correspondence: rubiodecasas@ugr.es
[3]Departamento de Ecología, Facultad de Ciencias, Universidad de Granada, 18071 Granada, Spain
Full list of author information is available at the end of the article

S1 Generalization of the model: absence of an explicit association between dispersal and mating

The models presented in the main text are based on a perfect correlation between dispersal and the mating system, as only non-dispersing seeds experience inbreeding depression. In this section, we show that this condition can be relaxed, leading to qualitatively similar conclusions.

In a more general scenario, we suppose that: (i) inbreeding modifies the *quality* of seeds, q, regardless of their dispersal strategy, which modulates their establishment probability $p_{\text{ext}}(t) \to q \times p_{\text{ext}}(t)$ and $p_{\text{int}} \to q \times p_{\text{int}}$, for dispersing and non-dispersing seeds respectively; (ii) we assume that mating is more likely to occur between individuals that are spatially close. Therefore, in the presence of inbreeding depression, the quality of the seeds produced by a plant is a function of its proximity to relatives and of its genetic similarity to these relatives, i.e., if a plant coexists with its close relatives, it is likely to mate with them and the resulting seeds are expected to carry a genetic load that reduces their quality. Assuming inbreeding depression is the product of multilocus interactions among many ($n \to \infty$) moderately or slightly deleterious alleles and that the number of said alleles that are homozygous in an inbred genotype will determine its fitness [1], the effect of inbreeding depression can be modeled as follows: given a plant i, we compute the quality of its seeds, $q(i)$, as:

$$q(i) = 1 - \left\langle e^{-a(i,j)/\delta'} \right\rangle_{j \in n.n.(i)} \tag{S1}$$

where $\langle \cdot \rangle_{j \in n.n.(i)}$ represents the average over the plant's immediate neighbors, δ' is the inbreeding depression parameter (see below), and $a(i,j)$ is the minimum number of past generations in which individuals i and j have a common ancestor, i.e., the degree of kinship between them, so that $a(i,j) = 1$ if they come from the same mother, $a(i,j) = 2$ for a common grandmother but different mothers, and so on. As eq. S1 is not defined in the case of a plant with zero neighbors, we take a maximum inbreeding depression that still can be modulated by δ', imposing for such case $q(i) = 1 - e^{-0.5/\delta'}$. This means that in the absence of neighbors, selfing is complete and homozigosity maximal. However, if any neighbor is present cross-pollination is assumed, regardless of the kinship between individuals.

Eq. S1 affects both dispersing and non-dispersing seeds, and therefore there is no assumed correlation between mating and dispersal. However, non-dispersing seeds

produce plants that are more likely to coexist with their relatives, and, indirectly, become more affected by inbreeding than those individuals coming from dispersed seeds.

To understand the role of the new parameter δ', we can analyze the extreme cases $\delta' \ll 1$ and $\delta' \gg 1$. For low values of δ', the exponential function in eq. S1 rapidly decreases to 0, and $q \to 1$; this limit represents a situation with very mild inbreeding depression. On the other hand, high values of δ' represent the case in which individuals are subject to high inbreeding depression even when mating with distantly related partners, as $q \to 0$. In conclusion, δ' plays a similar role to the parameter δ –controlling the level of inbreeding depression– in the original model.

In order to determine the optimal strategy, we also developed –as in the main text– an evolutionary implementation of the new model. Fig. S1 shows the results obtained by letting the mean dispersal propensity parameter α self-tune dynamically in a community of individuals through evolutionary dynamics based on a genetic algorithm in which each individual inherits its mixed dispersal propensity parameter from its ancestor with a small Gaussian mutation, so the parameter α self-tunes to the optimal value [2, 3]. This figure is equivalent to Fig. 6 of the main text and shows the effect on population density of inbreeding depression and environmental variability (upper panel) and the corresponding dispersal ESS for each combination of parameters (lower panel). As observed in the original model, mixed syndromes are selected for in the proximity of the critical point and the dispersing syndrome is favored whenever the environmental variability is low (red region). However, in this case the ESS corresponds to mixed dispersal whenever environmental variability is $\sigma > 0.1$ (orange to light blue region) for any value of δ'. Generally speaking, the ESS values of α are lower in this case than in the simpler model because the inbreeding depression affects both dispersal and non-dispersal propagules and thus the quality of non-dispersing individuals is not so severely penalized compared to dispersing ones. In other words, although under this generalization inbreeding affects all individuals, its influence on dispersal is less significant. As a result, α is largely independent from δ' for any $\delta' \leq 0.25$. However, under high values of δ' we again observe that the populations surviving under stressful conditions exhibit mixed dispersal syndromes (low density populations near the critical line in Fig. S1). In spite of the large parameter region in which mixed dispersal constitutes the ESS, purely non-dispersing syndromes ($\alpha \simeq 0$) are still not selected for under any parameterization, as evidenced by the absence of dark blue regions in the lower panel of Fig. S1 .

It is worth noting that even though the generalized model presented here no longer assumes a perfect association between mating and dispersal, it still considers only the case of a discrete dispersal polymorphism (i.e. seeds are either dispersed or implanted locally). Additionally, only dispersed seeds are affected by environmental fluctuations. We leave for future research further generalizations of the model considering continuous rather than discrete dispersal kernels as well as more extensive parameterizations of environmental variability.

S2 Generalization of the model: partial recovery with outcrossing

In the versions of our model presented so far, the quality of seeds decreases after an inbreeding event. If the genetic load brought on by inbreeding is represented by δ,

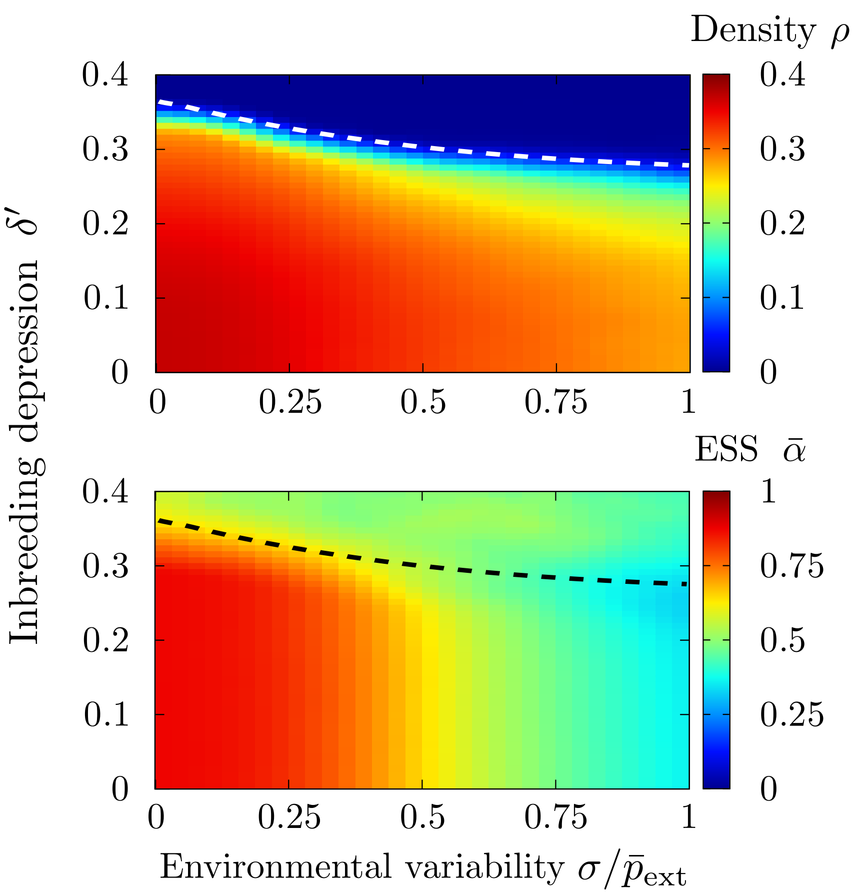

Supplementary Figure S1 Evolutionary stable strategies without an explicit association between dispersal and mating. (Upper panel) Stationary density ρ of the population as a function of the inbreeding depression parameter δ' and the environmental variability σ (relative to $\bar{p}_{\rm ext}$). (Lower panel) Average value of α across the population in its steady state. The dashed lines separate the absorbing from the active phase. Parameters have been set to $L = 100$, $n = 5$, $p_{\rm int} = \bar{p}_{\rm ext} = 0.25$ and $\nu = 10^{-2}$, and averages are performed over the last 5×10^3 steps in simulations iterated for 10^4 generations.

at generation t of inbreeding, $q(t+1) = (1-\delta)q(t)$. On the other hand, the quality factor is reseted to its maximum value, $q = 1$, following a single outcrossing event. This hypothesis can be relaxed by considering the case in which the quality is not fully restored after a single outcrossing event, but that recovers gradually. This can represented expressing the quality of dispersed seeds as $q(t+1) = q(t)+\beta(1-q(t))$, where β represents the *recovery* fraction parameter. Notice that $\beta = 1$ corresponds to the case studied so far while $\beta = 0$ describes a situation without recovery (for which the only stable solution after inbreeding is extinction).

As for other versions of the model, here we also compute the ESS by using a genetic algorithm. Fig. S2 shows the stationary density of the population, ρ (left panels), and the mean dispersal propensity ESS, $\bar{\alpha}$ (right panels), as a function of inbreeding depression parameter δ and environmental variability σ. Each row corresponds to a different value of the recovery fraction β (the case $\beta = 1$ corresponds to Fig. 6 in the main text).

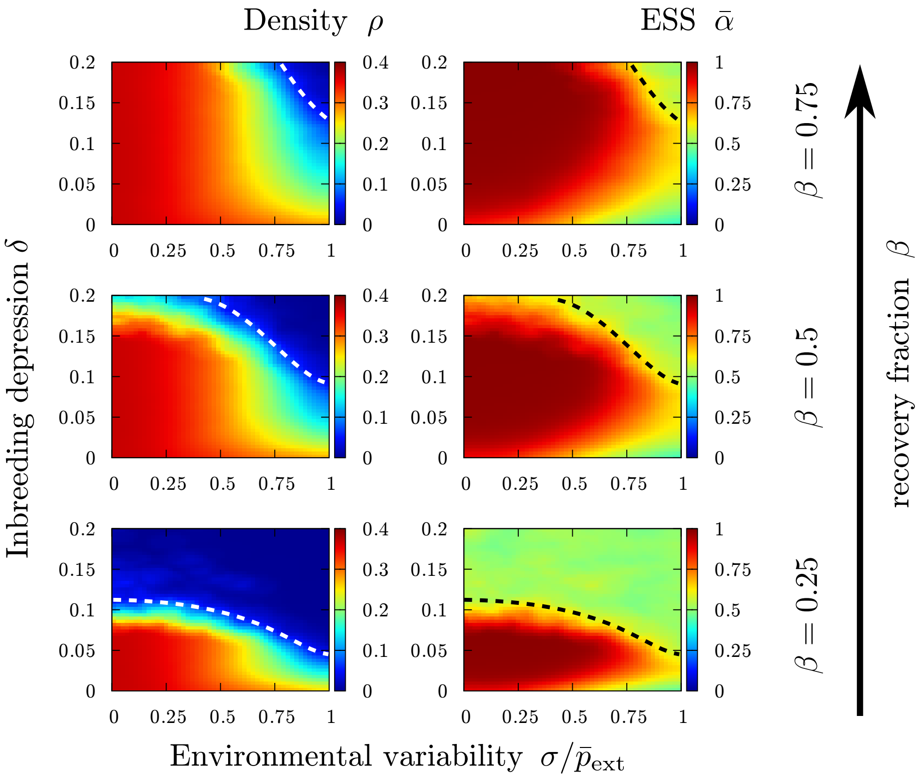

Supplementary Figure S2 ESS with partial recovery from inbreeding. These plots show the effect of partial recovery from inbreeding depression following outcrossing events. The recovery fraction is expressed by the parameter β (note that $\beta = 1$ corresponds to full recovery after a single outcrossing event, Fig. 6 in the main text). The panels represent results for the stationary population density ρ (left column) and the mean dispersal propensity $\bar{\alpha}$ in the ESS, under different values of β, inbreeding depression δ and environmental variability σ (relative to $\bar{p}_{\rm ext}$). The dashed lines separate the absorbing from the active phase. Parameters have been set to $L = 100$, $n = 5$, $p_{\rm int} = \bar{p}_{\rm ext} = 0.25$ and $\nu = 10^{-3}$, and averages are performed over the last $10^5/2$ steps in simulations iterated for 10^5 generations.

The absorbing region (i.e., the parameter space where $\rho = 0$) increases as the recovery fraction β becomes smaller. For all values of $\beta > 0$, mixed dispersal strategies seem to provide the ESS, even ir their region in parameter space shrinks as β decreases.

S3 Generalization of the model: sensitivity of the non-dispersing syndrome to environmental fluctuations

All the scenarios considered so far have been modeled assuming that non-dispersal provides a perfect buffer from environmental fluctuations. In this section, we explore the possibility that environmental variability also modulates the establishment probability of non-dispersed seeds.

The establishment probability for dispersing seeds at each generation, t, can be written as $p_{\rm ext}(t) = \bar{p}_{\rm ext} + \sigma\xi(t)$, where $\xi(t)$ is a uniform random variable in the interval $[0, 1]$. Similarly, we introduce environmental variability to the establishment probability of non-dispersing seeds, $p_{\rm int} \to p_{\rm int}(t) = \bar{p}_{\rm int} + \gamma\sigma\xi(t)$, where $\bar{p}_{\rm int}$ represents its mean value (taken to be the value calculated by the model without variation, $\bar{p}_{\rm int} = p_{\rm int}$), and where the parameter γ quantifies the *sensitivity* of the

non-dispersing syndrome to environmental fluctuations. In the limit $\gamma \to 0$, we recover the original model in which $p_{\rm int}(t)$ is a constant. Conversely, when $\gamma = 1$, non-dispersing seeds are not at all buffered from environmental variations.

Note that $\xi(t)$ is the same for $p_{\rm ext}(t)$ and $p_{\rm int}(t)$, as the environment affects dispersing and non-dispersing strategies simultaneously and in the same way (i.e., "seasons" will be favorable or unfavorable for both types of seeds). More general parameterizations could introduce different correlations between the establishment probabilities $p_{\rm ext}(t)$ and $p_{\rm int}(t)$ (even anti-correlations), but here we restrict our analysis to the perfectly correlated case. In order to keep establishment probabilities in the range $[0, 1]$, we take the environmental variability to lie in the interval $\sigma \leq \min(\bar{p}_{\rm int}, 1 - \bar{p}_{\rm int}, \bar{p}_{\rm ext}, 1 - \bar{p}_{\rm ext})$.

As in the other versions of our model, we computed the optimal strategy with a genetic algorithm in which the mean dispersal propensity parameter self-tunes dynamically. Fig. S3 represents the stationary density, ρ (left panels), and the dispersal propensity ESS, $\bar{\alpha}$ (right panels), as a function of the inbreeding depression and environmental variability, for different values of the sensitivity parameter $\gamma = 0.25, 0.5, 0.75, 1$ (note that $\gamma = 0$ corresponds to Fig. 6 in th main text).

Under moderate values of γ, results are largely similar to those obtained in the simpler model; mixed syndromes leading to viable populations (i.e. where $\rho > 0$) emerge for low values of inbreeding depression and high environmental variability. However, as sensitivity of the non-dispersing seeds to environmental fluctuations increases, we observe that: i) The region of parameters in which $\rho > 0$ is reduced, and ii) purely dispersing syndromes ($\alpha = 1$) tend to predominate. In other words, when non-dispersing provides little or no buffer from environmental variability, non-dispersing and dispersing syndromes share similar risks, but the former are also burdened by inbreeding depression, and therefore selection will necessarily favor dispersal. In conclusion, mixed syndromes are selected for under high values of environmental variability and low inbreeding depression as long as there is a trade-off between dispersing and non-dispersing in terms of risk (i.e. $\gamma < 1$).

Author details

[1]Instituto Carlos I de Física Teórica y Computacional and Departamento Electromagnetismo y Física de la Materia, Universidad de Granada 18071 Granada, Spain. [2]Dipartimento di Fisica 'G. Galilei' and CNISM, INFN, Universitá di Padova, Via Marzolo, 8, 35131 Padova, Italy. [3]Departamento de Ecología, Facultad de Ciencias, Universidad de Granada, 18071 Granada, Spain. [4]Estación Experimental de Zonas Áridas, EEZA-CSIC, Carretera de Sacramento s/n, La Cañada de San Urbano, 04120 Almería, Spain. [5]UMR 5175 Centre Ecologie Fonctionnelle et Evolutive, CEFE-CNRS, 1919 Route de Mende, 34293 Montpellier cedex 05, France.

References

1. Charlesworth, D., Willis, J.H.: The genetics of inbreeding depression. Nature Reviews Genetics **10**(11), 783–796 (2009)
2. Goldberg, D.E.: Genetic Algorithms in Search, Optimization, and Machine Learning. Addison-Wesley Professional, Boston (1989)
3. Gros, C.: Complex and Adaptive Dynamical Systems: A Primer. Springer, Berlin–Heidelberg (2013)

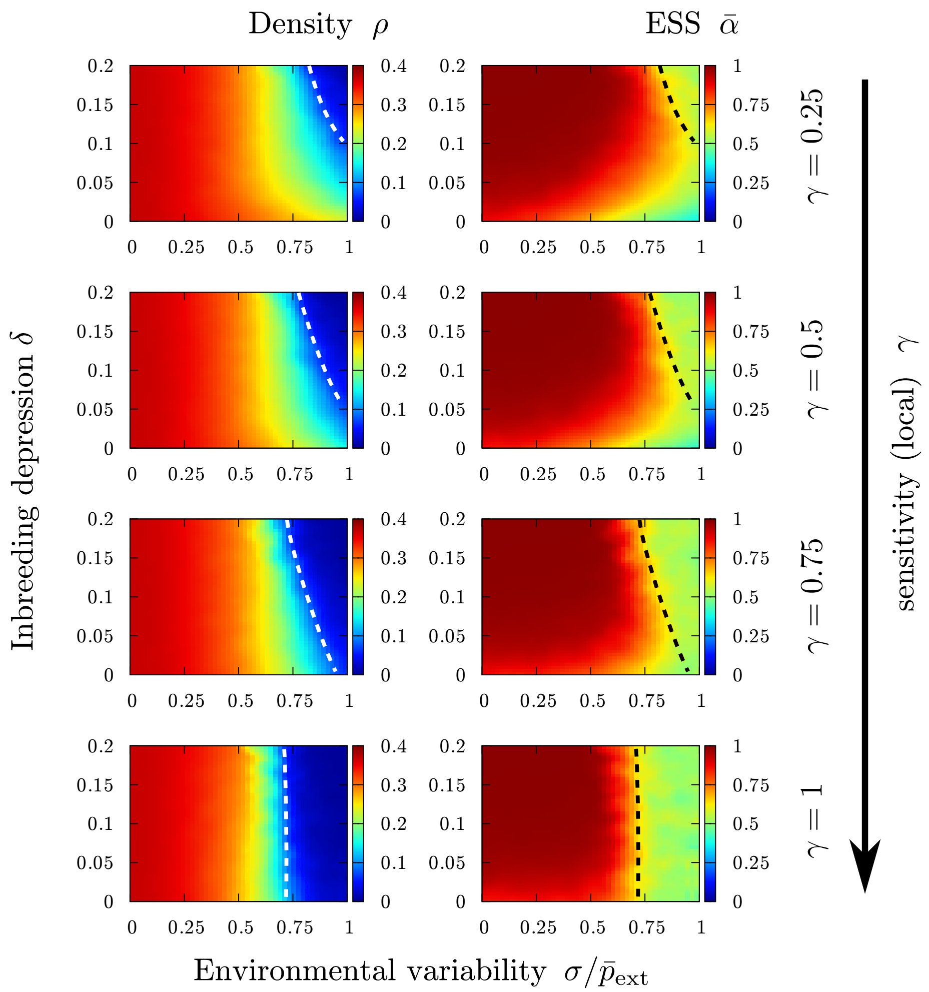

Supplementary Figure S3 ESS when environmental fluctuations affect both dispersal syndromes. For this calculations, the influence of environmental fluctuations on the quality of non-dispersing seeds has been approximated by a sensitivity parameter γ. Higher values of γ represent less buffering from environmental heterogeneity, whereas $\gamma = 0$ corresponds to the original case, Fig. 6 in the main text. Plots represent the results of simulations under different values of γ, δ and σ (relative to $\bar{p}_{\text{ext}}$). (Left panels) Stationary density of the population, (Right panels) dispersal propensity ESS ($\bar{\alpha}$). Parameters have been set to $L = 100$, $n = 5$, $\bar{p}_{\text{int}} = \bar{p}_{\text{ext}} = 0.25$ and $\nu = 10^{-3}$, and averages are performed over the last $10^5/2$ steps in simulations iterated for 10^5 generations.
