# Supplementary material for: Environmental unpredictability and inbreeding depression select for mixed dispersal syndromes
Source: BMC Evol Biol. 2016 Apr 5;16:71. doi: 10.1186/s12862-016-0638-8 (PMC4820946; doi:10.1186/s12862-016-0638-8)
Supplement: Additional file 2 — Appendix. Analytical approach for the model. (PDF 216 kb) [file 12862_2016_638_MOESM2_ESM.pdf]

RESEARCH

Appendix for: Environmental unpredictability and inbreeding depression select for mixed dispersal syndromes

Jorge Hidalgo[1,2], Rafael Rubio de Casas[3,4,5*] and Miguel Á. Muñoz[1]

*Correspondence: rubiodecasas@ugr.es
[3]Departamento de Ecología, Facultad de Ciencias, Universidad de Granada, 18071 Granada, Spain
Full list of author information is available at the end of the article

Analytical approach

In addition to the computational results described in the main text, we developed a mathematical model in order to better understand the mechanisms by which mixed dispersal affected population density and extinction time. This approach is, in essence, an analytical evaluation of the population average exponential growth rate of the computational model. This growth rate can be defined as:

$$G = \lim_{t\to\infty} \frac{1}{t} \log \frac{N(t)}{N(0)}. \tag{A1}$$

This quantity is negative for shrinking populations, positive for growing ones, or zero in the marginal or critical case; therefore, the sign of G can be regarded as an indicative proxy of the fate of the population. We calculated G as a function of the environmental and inbreeding parameters for different types of homogeneous populations with a fixed value of α: i) Single phenotype dispersing syndrome ($\alpha = 1$), ii) Single phenotype non-dispersing syndrome ($\alpha = 0$) and iii) mixed syndromes ($0 < \alpha < 1$). The mathematical analyses can be performed under the following approximations:

- Saturation effects –which become important at large densities preventing the population from growing infinitely– are neglected. Therefore, the following calculations provide valuable information only for the dynamics of low-density populations, allowing in particular the determination of critical points, but not to study stationary states.
- System sizes are sufficiently large, so that, statistical deviations from mean values can be safely neglected.
- Spatial correlations are not included; i.e. all sites are assumed for simplicity to be nearest neighbors.

In the terminology of statistical mechanics these approximations taken together constitute a linearized "mean field" approach [1, 2].

Dispersal syndrome ($\alpha = 1$)

In this approximation, $np_{\text{ext}}(t)$ is the average number of established seeds per plant at any given generation t. Thus, starting from a population $N(t = 0)$ individuals

after t generations the population size becomes

$$N(t) = np_{\text{ext}}(t)N(t-1) = ... = \left(\prod_{i=1}^{t} np_{\text{ext}}(i)\right) N(0). \tag{A2}$$

Using this expression and the definition of G,

$$G = \frac{1}{t}\sum_{i=1}^{t} \log\left(np_{\text{ext}}(i)\right) = \left\langle \log\left(np_{\text{ext}}(t)\right)\right\rangle_t , \tag{A3}$$

where $\langle\cdot\rangle_t$ represents the temporal average over generations using the probability distribution of $p_{\text{ext}}(t)$. Observe that, as

$$\frac{1}{t}\sum_{i=1}^{t} \log\left(np_{\text{ext}}(i)\right) = \log(\Pi_{i=1}^{t}\left(np_{\text{ext}}(i)\right)^{1/t}), \tag{A4}$$

the overall growth rate coincides with the geometric mean of growth rates across generations; i.e. population growth rate is a multiplicative process [3].

In the particular case in which p_{ext} is uniformly distributed in the range $[\bar{p}_{\text{ext}} - \sigma, \bar{p}_{\text{ext}} + \sigma]$ (and assuming that all possible values of p_{ext} have been homogeneously sampled for sufficiently large times t), G can be explicitly calculated as

$$G \;=\; \int_{\bar{p}_{\text{ext}}-\sigma}^{\bar{p}_{\text{ext}}+\sigma} dp_{\text{ext}} \frac{1}{2\sigma}\log\left(np_{\text{ext}}\right) \frac{1}{2\sigma}\log\left(n^{2\sigma}\frac{(\bar{p}_{\text{ext}}+\sigma)^{\bar{p}_{\text{ext}}+\sigma}}{(\bar{p}_{\text{ext}}-\sigma)^{\bar{p}_{\text{ext}}-\sigma}}\right) - 1, \tag{A5}$$

valid for $\sigma < p_{\text{ext}}$, while for the case in which $\sigma = p_{\text{ext}}$ the integral gives:

$$G = \log(2n\bar{p}_{\text{ext}}) - 1. \tag{A6}$$

Because the critical regime separates positive from negative population growth rates, it can be determined by solving the integral for $G = 0$. The resulting equations describing the critical regime as a function of σ and $\bar{p}_{\text{ext}}$ can then be solved numerically. These solutions are plotted as a dashed line in Fig. 3 of the main text. In the case where $\sigma = \bar{p}_{\text{ext}}$, the critical point is located at $\bar{p}^{c}_{\text{ext}} = e/2n$ (in our simulations we take $n = 5$, and therefore $\bar{p}^{c}_{\text{ext}} \simeq 0.27$).

Non-dispersal syndrome ($\alpha = 0$)

As, in this case, all seeds are inbred, their quality parameter q is reduced by a factor $(1-\delta)$ in each generation; i.e.

$$q(t) = (1-\delta)q(t-1) = ... = (1-\delta)^{t} q(0). \tag{A7}$$

As the establishment probability is $p_{\text{int}} \times q(t)$, a dramatic (exponential) reduction of this probability can be expected in time for any inbreeding penalty factor $\delta > 0$. In particular, assuming that all individuals in the community start with a common

quality $q(0)$ and taking into account that $1+2+...+t-1 = t(t-1)/2$ the population size at generation t is:

$$\begin{aligned} N(t) &= nq(t-1)p_{\rm int}N(t-1) = \left(\prod_{i=1}^{t} n(1-\delta)^{i-1}q(0)p_{\rm int}\right)N(0) \\ &= (1-\delta)^{t(t-1)/2}\left(nq(0)p_{\rm int}\right)^t N(0). \end{aligned} \tag{A8}$$

From this equation, G can be expressed as

$$G = \lim_{t\to\infty} \log\left((1-\delta)^{(t-1)/2} nq(0)p_{\rm int}\right). \tag{A9}$$

Note that, for any $\delta > 0$, we always find that $G < 0$ as $\lim_{t\to\infty} G = -\infty$. Instead, in the absence of inbreeding depression ($\delta = 0$), the critical transition point, $G = 0$, is found at $p^c_{\rm int} = (nq(0))^{-1}$ (see the inset of Fig. 3 in the main text).

Mixed dispersal syndrome ($0 < \alpha < 1$)

The calculation for mixed dispersal strategies is slightly more complicated than the two previous single-phenotype cases. For mixed dispersal, the quality parameter becomes a stochastic variable. The quality of non-dispersing seeds can be multiplicatively reduced sequentially (as for the $\alpha = 0$ case above); however, in the case of mixed strategies, the quality parameter is reset to one whenever seeds are dispersed. Despite the complexity of these dynamics, the distribution of quality parameters in the community, $\mathcal{P}(q,t)$, reaches a stationary state after a sufficiently large number of generations. A practical way to estimate the value of the stationary quality parameter consists in computing its mean value, $\bar{q}$, over individuals and generations. To do that we fix it self-consistently by imposing its mean value to remain unaltered from one generation to the next.

Defining $N^{\alpha=0}(t)$ and $N^{\alpha=1}(t)$ as the number of individuals grown from non-dispersed and dispersed seeds at generation t, respectively, the stationary quality can be expressed mathematically as

$$\bar{q} = \left\langle 1\cdot\frac{N^{\alpha=1}(t)}{N(t)} + (1-\delta)\bar{q}\frac{N^{\alpha=0}(t)}{N(t)}\right\rangle_t = \left\langle \frac{\alpha np_{\rm ext}(t) + (1-\alpha)np_{\rm int}(1-\delta)\bar{q}^2}{\alpha np_{\rm ext}(t) + (1-\alpha)np_{\rm int}\bar{q}}\right\rangle_t, \tag{A10}$$

This equation can be solved –even if implicitly– for $\bar{q}$ as a function of the dynamical parameters. In the case where $p_{\rm ext}(t)$ is uniformly distributed in the range $[\bar{p}_{\rm ext} - \sigma, \bar{p}_{\rm ext} + \sigma]$, and assuming that time t is large enough as to homogeneously sample all values of the $p_{\rm ext}$ distribution, the average quality can be calculated as:

$$\bar{q} = 1 + \frac{1}{2\sigma\alpha}p_{\rm int}\left(1-(1-\delta)\bar{q}\right)\bar{q}(1-\alpha) = \log\frac{p_{\rm int}\bar{q}(1-\alpha) + \alpha(\bar{p}_{\rm ext}-\sigma)}{p_{\rm int}\bar{q}(1-\alpha) + \alpha(\bar{p}_{\rm ext}+\sigma)}. \tag{A11}$$

The numerical solution of eq. A11 is represented in Fig. A1 (upper panel) as a function of the parameter α for different values of inbreeding depression δ. The numerical solutions are presented together with the comparable computational approximations. This figure shows that, as expected after multiple generations $\bar{q}(\alpha = 0) = 0$

and $\bar{q}(\alpha = 1) = 1$ corresponding to the quality of populations with single non-dispersal and dispersal syndromes respectively. We can see that, although we have used a simplistic approximation, the computed $\bar{q}$ constitutes a good estimate of the actual value determined from computer simulations of the full model.

Using the inferred value of $\bar{q} = \bar{q}(\alpha, \bar{p}_{\text{ext}}, \sigma, p_{\text{int}}, \delta)$, we can compute $N(t)$:

$$N(t) = (\alpha n p_{\text{ext}}(t) + (1-\alpha) n \bar{q} p_{\text{int}})\, N(t-1) = ... = \prod_{i=1}^{t} (\alpha n p_{\text{ext}}(i) + (1-\alpha) n \bar{q} p_{\text{int}})\, N(0), \tag{A12}$$

and from this

$$G = \langle \log (\alpha n p_{\text{ext}}(t) + (1-\alpha) n \bar{q} p_{\text{int}}) \rangle_t \,, \tag{A13}$$

which in the case of a uniformly distributed environment in $[\bar{p}_{\text{ext}} - \sigma, \bar{p}_{\text{ext}} + \sigma]$ becomes

$$G = -1 + \frac{1}{2\sigma\alpha} \log \left(n^{2\sigma\alpha} \frac{[(1-\alpha)\bar{q}p_{\text{ext}} + \alpha(\bar{p}_{\text{ext}} + \sigma)]^{(1-\alpha)\bar{q}p_{\text{ext}} + \alpha(\bar{p}_{\text{ext}} + \sigma)}}{[(1-\alpha)\bar{q}p_{\text{ext}} + \alpha(\bar{p}_{\text{ext}} - \sigma)]^{(1-\alpha)\bar{q}p_{\text{ext}} + \alpha(\bar{p}_{\text{ext}} - \sigma)}} \right) \tag{A14}$$

where $\bar{q} = \bar{q}(\alpha, \bar{p}_{\text{ext}}, \sigma, p_{\text{int}}, \delta)$ is the solution of eq. A11. In Fig. A1 (lower panel) the growth rate is plotted as a function of the dispersal fraction α, for the same choice of parameters in Fig. 5 in the main text, i.e. when both dispersing and non-dispersing syndromes are nonviable ($G(\alpha = 0, 1) < 0$). As a consequence of its parabolic-like shape, G intersects zero and becomes positive for intermediate values of the dispersal fraction. Therefore, the analytical prediction confirms that even when populations exhibiting either of the single phenotypes are bound to collapse, mixed dispersal syndromes can allow for long term population stability.

Author details
[1]Instituto Carlos I de Física Teórica y Computacional and Departamento Electromagnetismo y Física de la Materia, Universidad de Granada 18071 Granada, Spain. [2]Dipartimento di Fisica 'G. Galilei' and CNISM, INFN, Universitá di Padova, Via Marzolo, 8, 35131 Padova, Italy. [3]Departamento de Ecología, Facultad de Ciencias, Universidad de Granada, 18071 Granada, Spain. [4]Estación Experimental de Zonas Áridas, EEZA-CSIC, Carretera de Sacramento s/n, La Cañada de San Urbano, 04120 Almería, Spain. [5]UMR 5175 Centre Ecologie Fonctionnelle et Evolutive, CEFE-CNRS, 1919 Route de Mende, 34293 Montpellier cedex 05, France.

References

1. Marro, J., Dickman, R.: Nonequilibrium Phase Transitions in Lattice Models. Cambridge University Press, Cambridge (2005)
2. Grinstein, G., Muñoz, M.: The statistical mechanics of absorbing states. In: Fourth Granada Lectures in Computational Physics, pp. 223–270. Springer, Berlin (1997)
3. Dempster, E.R.: Maintenance of genetic heterogeneity. In: Cold Spring Harbor Symposia on Quantitative Biology, vol. 20, pp. 25–32 (1955). Cold Spring Harbor Laboratory Press

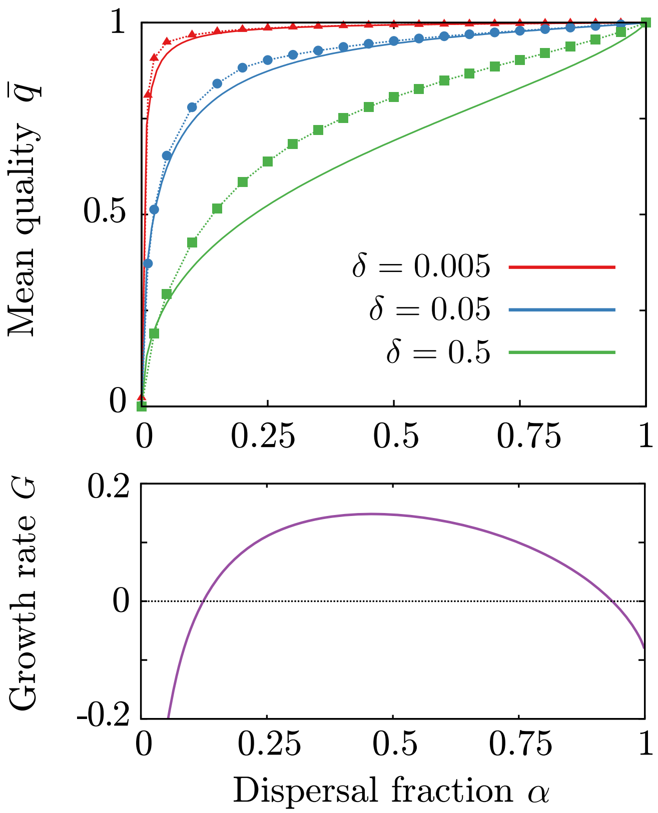

Figure A1 (Upper panel) Results of the self-consistent calculation used to estimate the averaged quality factor of seeds in a population with mixed dispersal strategies. After a sufficiently large number of generations, the quality parameter q converges, on average, to a steady state value $\bar{q}$, which depends on α as shown for three different values of the inbreeding depression parameter δ. The approximate values obtained within the self-consistent calculation (solid lines) are not far from the computational measured results (colored points), indicating that the approximations are sound. Observe that in the limit $\delta \to 0$ the curves converge to $\bar{q} = 1$ as expected. **(Lower panel) Exponential growth rate G as a function of the dispersal propensity parameter** α. The growth rate, given by eq. A14, interpolates non-linearly between $G(\alpha = 0, \delta > 0) = -\infty$ (eq. A9) and $G(\alpha = 1)$ (eq. A5). As a consequence of its parabolic-like shape, $G(\alpha)$ intersects at zero, and intermediate values of the dispersal propensity parameter, α can be associated with $G(\alpha) > 0$, even if $G(\alpha = 0, 1) < 0$ (extinction); i.e., mixed dispersal can allow large populations to survive indefinitely. Parameters are as in Fig. 5 of the main text: $p_{\rm int} = \bar{p}_{\rm ext} = \sigma = 0.25$, $n = 5$ and, in the lower panel, $\delta = 0.05$. Each point was computed in a community of $L = 100$, averaging over the last $10^5/2$ generations of 10 independent simulations iterated for 10^5 steps.
